# King cobra peptide OH-CATH30 as a potential candidate drug through clinic drug-resistant isolates

**DOI:** 10.24272/j.issn.2095-8137.2018.025

**Published:** 2018-03-07

**Authors:** Feng Zhao, Xin-Qiang Lan, Yan Du, Pei-Yi Chen, Jiao Zhao, Fang Zhao, Wen-Hui Lee, Yun Zhang

**Affiliations:** 1 Key Laboratory of Subtropical Medicinal Edible Resources Development and Utilization in Yunnan Province, Department of Biology and Chemistry, Puer University, Puer Yunnan 665000, China; 2 Key Laboratory of Bioactive Peptides of Yunnan Province/Key Laboratory of Animal Models and Human Disease Mechanisms of Chinese Academy of Sciences, Kunming Institute of Zoology, Kunming Yunnan 650223, China; 3 Department of Clinical Laboratories, First Affiliated Hospital of Kunming Medical University, Kunming Yunnan 650032, China; 4 Institute of Comparative Study of Traditional Materia Medica, Institute of Integrative Medicine of Fudan University, Shanghai 200433, China

**Keywords:** Cathelicidin, Antibacterial agent, Clinical isolates, OH-CATH30

## Abstract

Cationic antimicrobial peptides (AMPs) are considered as important candidate therapeutic agents, which exert potent microbicidal properties against bacteria, fungi and some viruses. Based on our previous findings king cobra cathelicidin (OH-CATH) is a 34-amino acid peptide that exerts strong antibacterial and weak hemolytic activity. The aim of this research is to evaluate the efficacy of both OH-CATH30 and its analog D-OH-CATH30 against clinical isolates comparing with routinely utilized antibiotics *in vitro*. In this study, 584 clinical isolates were tested (spanning 2013–2016) and the efficacy of the candidate peptides and antibiotics were determined by a broth microdilution method according to the CLSI guidelines. Among the 584 clinical isolates, 85% were susceptible to OH-CATH30 and its analogs. Both L- and D-OH-CATH30 showed higher efficacy against (toward) Gram-positive bacteria and stronger antibacterial activity against nearly all Gram-negative bacteria tested compare with antibiotics. The highest bactericidal activity was detected against *Acinetobacter spp.*, including multi-drug-resistant *Acinetobacter baumannii* (MRAB) and methicillin-resistant *Staphylococcus aureus* (MRSA). The overall efficacy of OH-CATH30 and its analogs was higher than that of the 9 routinely used antibiotics. OH-CATH30 is a promising candidate drug for the treatment of a wide variety of bacterial infections which are resistant to many routinely used antimicrobial agents.

## INTRODUCTION

The abundant use of traditional antibiotics has resulted in the emergence of many antibiotic-resistant isolates worldwide, causing threatening to human health and the rate of antibiotic discovery and production has been insufficient to respond to this crisis (Fischbach & Walsh, 2009; Nathan, 2004). Recent studies have shown that there are many components in the venom that possess antimicrobial activity. Snake venom, produced by specialized glands in the snake jawbone, is a particularly rich source of such antimicrobial compounds (Tashima et al., 2012). Several venom compounds have been used for antimicrobial effects, such as phospholipases A2, metalloproteinases, L-amino acid oxidases and antimicrobial peptides (De Oliveira Junior et al., 2013). Cathelicidins have been identified as the main family of naturally-occurring antimicrobial peptides from snake venom, which exhibit potential microbicidal properties against bacteria, fungi and some viruses (Zhang, 2015). Currently, at least 9 cathelicidin-type antimicrobial peptides have been identified from elapid snake venoms, most of which exhibit potential antimicrobial activities (Falcao et al., 2014; Zhao et al., 2008). Cationic antimicrobial peptides (AMPs) are considered as important candidate therapeutic agents comprising a diverse group of bactericidal molecules for which microbial organisms show lower levels of resistance, for example pexiganan, an AMP developed for the treatment of diabetic foot infection (Ge et al., 1999). Although several peptide-antibiotics are well established clinically, such as polymixin B, AMPs alone without any modifications have not been widely used in clinical treatment (Hancock & Sahl, 2006). As an unique family of AMPs, the cathelicidin peptides shared conserved N-terminal domains from a variety of species and exhibited effective antimicrobial activity and some cytotoxic activity towards eukaryotic cells (Johansson et al., 1998; Zanetti et al., 1995).

**Figure 1:**
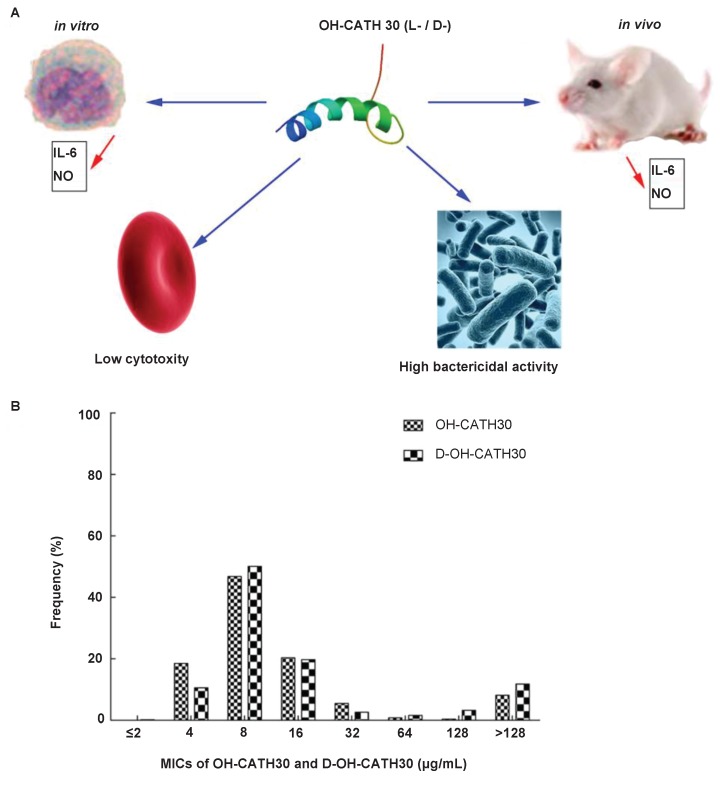
Cytotoxicity of OH-CATH30 and its analog

We previously reported a novel cathelicidin-derived peptide from the king cobra. This reptile cathelicidin was termed OH-CATH30 and exhibited potential broad-spectrum antibacterial activity *in vitro* and relatively low toxicity *in vivo* (Li et al., 2012; Zhang et al., 2010). The aim of this study is to evaluate the efficacy of OH-CATH30 and its analog D-OH-CATH30 (comprising all D-amino acids residues) against clinical isolates (collected from patients in hospital) compared with routinely utilized antibiotics *in vitro*. Meanwhile, the corresponding information is crucial for the cytotoxicity to eukaryotes and the immunogenicity of these peptides, which hemolysis assay and IL-6 assay were further investigated. Considering that most of AMPs have free radical scavenging ability, we also expanded nitric oxide assay and detected the release of nitric oxide (Figure 1A).

A: The workflow of cytotoxicity determination to L-OH-CATH30 and D-OH-CATH30; B: Distribution of L-OH-CATH30 and D-OH-CATH30 MICs for 584 bacterial isolates.

## MATERIALS AND METHODS

### Ethics statement

This study was approved by the Ethics Committee of Puer University and the Biomedical Ethics Committee of the First Affiliated Hospital of Kunming Medical University. The data were analyzed anonymously, patient identity deduced or disclosed.

### Clinical isolates, reference strains and materials

A total of 584 clinical isolates were collected between July 2013 and June 2016, which included 14 different species as follows: (i) *Acinetobacter spp.* (*n=*53); (ii) *Citrobacter spp.* (*n=*7); (iii) *Enterobacter spp.* (*n=*41); (iv) *Escherichia spp.* (*n=*229); (v) *Klebsiella spp.* (*n=*126); (vi) *Proteus spp.* (*n=*4); (vii) *Pseudomonas spp.* (*n=*76); (viii) *Salmonella spp.* (*n=*16); (ix) *Serratia spp.* (*n=*12); (x) *Sphingobacterium spiritivorum* (*n=*1); (xi) *Staphylococcus aureus* (MSSA) (*n=*2) and *S. aureus* (MRSA) (*n=*2); (xii) *Stenotrophomonas maltophilia* (*n=*9); (xiii) *Streptococcus pneumoniae.* (*n=*2); and (xiv) *Yersinia spp.* (*n=*4). The species identification of each clinical isolates was confirmed with the Vitek 2 system (bioMérieux, France; part of the data shown in Supplementary Figure S1). The antibiotic susceptibility of the clinical isolates was determined using the Kirby-Bauer disk diffusion method, in accordance with the Clinical and Laboratory Standards Institute (CLSI) 2009 guidelines (Clinical and Laboratory Standards Institute, 2009). Twelve control strains were also included: *E. coli* ATCC 25922 and ATCC 35218, *Haemophilus influenza* ATCC 49766 and ATCC 49247, *S. aureus* ATCC 25923 and ATCC 43300, *Klebsiella pneumoniae* ATCC 13883 and ATCC 700603, *S. maltophilia* ATCC 8090, *Enterococcus faecalis* ATCC 29212, *Enterobacter cloacae* ATCC 13047 and *Pseudomonas aeruginosa* ATCC 27853. All strains were cultured in MHB Mueller-Hinton broth (MHB, pH 7.2) medium at 37 ℃. All other reagents were of analytical grade and were obtained from commercial sources.

### Antibiotics

Ampicillin (Amp) and cefoperazone (CPZ) were acquired from the General Pharmaceutical Factory of Harbin Pharmaceutical Group (Harbin, China). Roxithromycin (ROX), chloramphenicol (CHL) and azithromycin (AZM) were from Sangon Biotech Co., Ltd. (Shanghai, China). Amikacin (AMK), vancomycin (VAN), levofloxacin (LVX) and polymyxin (PB) were from Sigma-Aldrich Co. LLC (Sigma, St. Louis, MO, USA).

### Antimicrobial peptides synthesis

Pexiganan (GIGKFLKKAKKFGKAFVKILKK-NH2), OH-CATH30 (KFFKKLKNSVKKRAKKFFKKPRVIGVSIPF) and its analog D-OH-CATH30 (*KFFKKLKNSVKKRAKKFFKKPRVIGVSIPF*, italics indicate D-amino acids) were synthesized by solid-phase synthesis on an Applied Biosystems Model 433A peptide synthesizer according to the manufacturer’s standard protocols, as reported previously (Li et al., 2012). Then, the crude synthetic peptide was purified by reverse-phase high-performance lipid chromatography (RP-HPLC, Supplementary Figure S2). Mobile phase eluent A consisted of 0.1% TFA (aqueous) and mobile phase eluent B consisted of ACN/ddH2O/TFA 90/10/0.08% (v/v/v). The sample was added along with auto-sampler to a Thermo Scientific EASY loading column (Thermo Fisher Scientific, USA) (2 cm×100 μm, 5 μm-C18) then to an analytic column (75 μm×100 mm, 3 μm-C18) with a flow rate of 1.2 mL/min. The presence of peptides was confirmed by an absorption peak at 215 nm. The purity of each synthetic peptide was above 95%. The identity and purity of each peptide was further confirmed by matrix-assisted laser desorption ionization-time of flight (MALDI-TOF) mass spectrometry (Voyager; Supplementary Figure S2). The survey of the full scan MS spectra (m/z 300−1 800) was acquired in the Orbit rap with a 70 000-resolution (m/z 200). Dynamic exclusion was set to 15s. The 10 most intense multiply charged ions (z≥2) were sequentially isolated and fragmented by higher-energy collisional dissociation (HCD) with a fixed injection time of 80 ms and 17 500-resolution (m/z 200). The conditions were as follows: spray voltage, 2 kV; no sheath and auxiliary gas flow; heated capillary temperature, 250 °C; and normalized HCD collision energy 27 eV; underfill ratio 0.1%. The MS/MS ion selection threshold was set at 1×10^5^ counts.

### Determination of MICs

MICs were determined by the broth microdilution method (Wiegand et al., 2008) according to the CLSI guidelines (Clinical and Laboratory Standards Institute, 2009). In each well of a 96-well plate, 100 µL of MHB broth and bacteria (5×10^5^ colony-forming unit (cfu)/mL) were incubated with various concentrations of antimicrobials for 16 h at 37 °C. The concentrations of the antimicrobials were the same for each of the tested bacterial strains in three independent experiments, and the MIC values were obtained without inter-experiment variations and expressed as µg/mL. For compounds and antibiotics drugs, the interpretation criteria were set by using the CLSI published criteria. The concentration of antibiotics was initially set at 512 µg/mL, then diluted to 4 µg/mL. As for the (only two are listed) peptides used in this study (pexiganna, polymyxin B), the concentration was diluted from 256 µg/mL to 2 µg/mL. In each batch of susceptibility tests, the 12 control strains listed above were included as a quality control.

### Hemolytic assay and cytokine determination

Hemolytic assays were investigated using human red blood cells in liquid medium as previously reported (Yang et al., 2012). Serial dilutions of the samples were incubated with washed red blood cells (2%) at 37 °C for 30 min, the cells were then centrifuged, and the absorbance of the supernatant was measured at 540 nm. Maximum hemolysis was determined by adding 1% Triton X-100 to the cell samples (Liu et al., 2012). 

An ELISA kit (Neobiosoence) was employed for IL-6 determination according to the manufacturer’s instructions. Nitric oxide was quantified using the Griess chemical method (Park et al., 1993).

IL-6 detection *in vitro*: Prior to euthanization, mice were administered 3 mL of saline by intraperitoneal injection. The abdomen was kneaded for 2 min before being opened up and the peritoneal fluid was collected. After excision, the mice livers and kidneys were placed into physiological saline, cut(sliced) into pieces, homogenized with trypsin, ground into fluid, passed through a mesh sieve and centrifuged at 2 000 r/min for 10 min. The supernatant was discarded and the pellet was washed with PBS until the supernatant was clear. Then the cells were re-suspended in F12 medium and 190 μL of cells (about 1×10^7^) were added to each well of a 96-well plate before the adding 10 μL of different concentrations of OH-CATH30 as the experimental group or F12 medium as the control. The plate was then incubated at 36 ℃for 2 h, followed by centrifugation at 3 000 r/min for 20 min. The supernatant was retained for further determinations. 

IL-6 detection *in vivo*: Mice were intraperitoneally injected with 2 mL of saline, then after 5 h were intraperitoneally injected with 5 mg/kg or 10 mg/kg of different types of OH-CATH30 (Li et al., 2013). Mice were euthanized, then the abdomen was kneaded for 2 min, after which it was opened and the peritoneal fluid was collected. After excision, the livers and kidneys were placed into physiological saline, sliced into pieces, homogenized with trypsin, ground into fluid, passed through a mesh sieve and centrifuged at 3 000 r/min for 20 min. The supernatant was discarded and the pellet was washed with PBS until the supernatant was clear.

### Statistical analysis

The data was analyzed using two-way ANOVA. The experimental data are expressed as MIC_50_ and MIC_90_ values. The level of statistical significance was set at *P*<0.05.

## RESULTS

### Susceptibility test

The *in vitro* susceptibility of 584 clinical isolates to three AMPs and nine antibiotics was tested, which was also represented by its cytotoxicity of prokaryotes cell (Figure 1A). Table 1 shows the MICs, with ranges of MIC_50_ and MIC_90_ values, of each antimicrobial agent tested against 14 different species. Overall, the efficacy of OH-CATH30 and D-OH-CATH30 was higher when compared with 9 routine antibiotics, and slightly higher than pexiganan. Among the 584 clinical isolates tested, 85% were susceptible to OH-CATH30 and its analog with a MIC≤64 mg/L (Figure 1B). For *Escherichia* spp. and *Klebsiella* spp. (61% of all clinical isolates), OH-CATH30 was more active than the 9 antibiotics tested, and showed slightly higher activity than pexiganan. For 53 strains of *Acinetobacter* spp., including multidrug-resistant *Acinetobacter baumannii* (MRAB), the MIC_90_ value of D-OH-CATH30 was doubled comparing with that of OH-CATH30, and polymixin B also had high rates of susceptibility (77%) to *Acinetobacter* spp. compared with other antibiotics, except for pexiganan. For the 76 strains of *Pseudomonas* spp., the activity of OH-CATH30 and its analog was higher than pexiganan, although these peptides are all showed higher activity than the 9 antibiotics, with the exception of levofloxacin. For the 16 strains of *Salmonella* spp., all showed high susceptibility to the antimicrobial peptides and antibiotics, with the exception of ampicillin and cefoperazone. The rate of susceptibility (92%) to levofloxacin among these species was considerably higher than the other 8 antibiotics. Among the 14 species of clinical isolates tested, *Serratia* spp., for which there were 12 strains, had the lowest rate of susceptibility to antimicrobial peptides and the peptide-antibiotic (polymixin B). OH-CATH30 inhibited all of the species, comprising around 10 strains, at a concentration of ≤ 64 mg/L, with the exception of *Proteus* spp. and *S. maltophilia.* Of the four strains of *S. aureus* tested, including two MSSA and two MRSA, D-OH-CATH30 was found to be more effective at inhibiting MRSA than OH-CATH30, with a range of ≤ 8 mg/L. Overall, L- OH-CATH30 showed higher efficacy against two thirds of the tested clinical isolates than OH-CATH30. 

Table 1MICs of three antimicrobial peptides and nine antibiotics determined for 584 clinic isolates, and the MIC_50_ and MIC_90_ values

**Table:** 

Isolates	No.	OH-CATH30 MIC(mg/L)			D-OH-CATH30 MIC (mg/L)			Amp^b^ MIC (mg/L)			Percentage of isolates susceptible to^c^ (%)	CPZ^b^ MIC (mg/L)				Percentage of isolates susceptible to^c^ (%)
		MIC_50_^a^	MIC_90_^a^	Range	MIC_50_^a^	MIC_90_^a^	Range	MIC_50_^a^^, ***^	MIC_90_^a^^, ***^	Range		MIC_50_^a^^, ***^	MIC_90_^a^^, ***^	Range		
*Acinetobacter sp.*	53	16	32	8–>128	8	16	2–>128	>256	>256	8–>256	0	>256	>256	4–>256		2
*Citrobacter sp.*	7	8	16	4–>128	8	>128	4–>128	>256	>256	8–>256	20	>256	>256	4–>256		40
*Enterobacter sp.*	41	8	32	4–>128	8	>128	4–>128	>256	>256	64–>256	0	>256	>256	4–>256		33
*Escherichia sp.*	229	8	64	2–>128	8	>128	2–>128	>256	>256	8–>256	5	>256	>256	4–>256		25
*Klebsiella sp.*	126	8	64	4–>128	16	128	2–>128	>256	>256	16–>256	0	>256	>256	4–>256		29
*Proteus sp.*	4	>128	>128	8–>128	8	128	8–>128	>256	>256	16–>256	0	256	256	4–256		33
*Pseudomonas sp.*	76	8	128	4–>128	16	>128	4–>128	>256	>256	16–>256	5	256	>256	4–>256		23
*Salmonella sp.*	16	8	8	4–>128	4	>128	4–>128	64	>256	4–>256	54	32	>256	4–>256		54
*Serratia sp.*	12	>128	>128	4–>128	8	64	8–>128	>256	>256	128–>256	0	256	>256	4–>256		36
*Sphingobacterium spiritivorum*	1	8	8	8	16	16	16	>256	>256	>256	0	>256	>256	>256		0
*Staphylococcus aureus(MSSA)*^d^	2	16	16	16	32	64	32–64	>256	>256	>256	0	>256	>256	>256		0
*Staphylococcus aureus(MRSA)*^e^	2	16	32	16–32	8	8	8	>256	>256	>256	0	>256	>256	>256		0
*Stenotrophomonas maltophilia*	9	8	>128	4–>128	8	32	4–32	>256	>256	128–>256	0	256	>256	4–>256		13
*Streptococcus pneumoniae*	2	8	8	8	8	>128	8–>128	>256	>256	>256	0	>256	>256	>256		0
*Yersinia sp.*	4	8	>128	8–>128	8	>128	8–>128	>256	>256	>256	0	>256	>256	>256		0

**Table:** Continued Table 1.

Isolates	No.	ROX^b^ MIC (mg/L)			Percentage of isolates susceptible to^c^ (%)	AZM^b^ MIC (mg/L)			Percentage of isolates susceptible to^c^ (%)	AMK^b^ MIC (mg/L)			Percentage of isolates susceptible to^c^ (%)	PB^b^ MIC (mg/L)			Percentage of isolates susceptible to^c^ (%)
		MIC_50_^a^^, ***^	MIC_90_^a^^, ***^	Range		MIC_50_^a^	MIC_90_^a^^, ***^	Range		MIC_50_^a^	MIC_90_^a^^, *^	Range		MIC_50_^a^	MIC_90_^a^	Range	
*Acinetobacter sp.*	53	>256	>256	8–>256	N/A	>256	>256	4–>256	N/A	>256	>256	4–>256	28	<2	32	2–>128	77
*Citrobacter sp.*	7	>256	>256	32–>256	N/A	16	>256	4–>256	N/A	8	>256	4–>256	60	<2	>128	2–>128	100
*Enterobacter sp.*	41	>256	>256	128–.256	N/A	8	>256	4–>256	N/A	<4	>256	4–>256	67	<2	128	2–>128	69
*Escherichia sp.*	229	>256	>256	16–>256	N/A	8	128	4–>256	N/A	<4	>256	4–>256	66	<2	128	2–>128	78
*Klebsiella sp.*	126	256	>256	32–>256	N/A	8	>256	4–>256	N/A	<4	>256	4–>256	58	<2	>128	2–>128	74
*Proteus sp.*	4	>256	>256	>256	N/A	32	128	32–128	N/A	<4	4	4	100	<2	>128	2–>128	67
*Pseudomonas sp.*	76	128	>256	4–>256	N/A	64	>256	4–>256	N/A	8	>256	4–>256	54	<2	>128	2–>128	67
*Salmonella sp.*	16	128	256	128–>256	N/A	<4	64	4–64	N/A	<4	32	4–>256	69	<2	64	2–64	77
*Serratia sp.*	12	>256	>256	32–>256	N/A	16	>256	4–>256	N/A	<4	64	4–>256	73	>128	>128	2–>128	9
*Sphingobacterium spiritivorum*	1	>256	>256	>256	N/A	16	16	16	N/A	<4	<4	<4	100	<2	<2	<2	100
*Staphylococcus aureus(MSSA)*^d^	2	256	>256	256–>256	0	<4	16	<4–16	50	<4	<4	<4	100	4	>128	4–>128	0
*Staphylococcus aureus(MRSA)*^e^	2	>256	>256	>256	0	8	>256	8–>256	0	<4	<4	<4	100	8	8	8	0
*Stenotrophomonas maltophilia*	9	128	>256	8–>256	N/A	64	>256	4–>256	N/A	32	>256	4–>256	50	<2	64	2–64	88
*Streptococcus pneumoniae*	2	>256	>256	>256	0	8	>256	8–>256	0	<4	>256	<4–>256	100	<2	>128	<2–>128	50
*Yersinia sp.*	4	32	>256	32–>256	N/A	16	256	16–256	N/A	<4	64	4–64	100	<2	>128	2–>128	100

**Table:** Continued Table 1.

Isolates	No.	CHL^b^ MIC (mg/L)			Percentage of isolates susceptible to^c^ (%)	PEX^b^ MIC (mg/L)			Percentage of isolates susceptible to^c^ (%)	LVX^b^ MIC (mg/L)			Percentage of isolates susceptible to^c^ (%)	VAN^b^ MIC(mg/L)			Percentage of isolates susceptible to^c^ (%)
		MIC_50_^a^	MIC_90_^a^^, ***^	Range		MIC_50_^a^	MIC_90_^a^	Range		MIC_50_^a^^, *^	MIC_90_^a^^, ***^	Range		MIC_50_^a^	MIC_90_^a^^, ***^	Range	
*Acinetobacter sp.*	53	128	256	8–>256	5	8	16	4–128	56	128	>256	16–>256	N/A	128	256	8–>256	5
*Citrobacter sp.*	7	8	32	4–32	80	<4	32	4–32	60	256	>256	4–>256	N/A	8	32	4–32	80
*Enterobacter sp.*	41	16	>256	4–>256	53	<4	32	4–128	72	256	>256	64–>256	N/A	16	>256	4–>256	53
*Escherichia sp.*	229	<4	128	4–>256	58	<4	128	4–256	55	128	>256	8–>256	N/A	<4	128	4–>256	58
*Klebsiella sp.*	126	16	256	4–>256	46	>256	32	4–128	48	8	>256	8–>256	N/A	16	256	4–>256	46
*Proteus sp.*	4	8	256	8–256	67	8	16	4–128	33	128	256	128–256	N/A	8	256	8–256	67
*Pseudomonas sp.*	76	<4	>256	4–>256	68	<4	64	4–128	63	>256	>256	64–>256	N/A	<4	>256	4–>256	68
*Salmonella sp.*	16	<4	256	4–>256	84	<4	4	4–128	92	>256	>256	4–>256	N/A	<4	256	4–>256	84
*Serratia sp.*	12	64	>256	4–>256	0	<4	16	4–32	55	>256	>256	64–>256	N/A	64	>256	4–>256	0
*Sphingobacterium spiritivorum*	1	<4	<4	<4	100	<4	<4	<4	100	256	256	256	N/A	<4	<4	<4	100
*Staphylococcus aureus(MSSA)*^d^	2	<4	32	<4–32	50	8	8	8	0	<4	8	<4–8	50	<4	32	<4–32	50
*Staphylococcus aureus(MRSA)*^e^	2	<4	<4	<4	100	16	32	16–32	0	<4	>256	<4–>256	50	<4	<4	<4	100
*Stenotrophomonas maltophilia*	9	32	>256	4–>256	50	<4	256	4–256	63	128	>256	128–>256	N/A	32	>256	4–>256	50
*Streptococcus pneumoniae*	2	<4	<4	<4	100	16	32	16–32	0	<4	<4	<4	50	<4	<4	<4	100
*Yersinia sp.*	4	<4	<4	<4	100	<4	4	4	100	256	>256	4–>256	N/A	<4	<4	<4	100

^a^: MIC_50_ and MIC_90_, MICs at which 50%, or 90% of isolates are inhibited, respectively; ^b^: Amp, Ampicillin; CPZ, Cefoperazone; ROX, Roxithromycin; CHL, Chloramphenicol; AZM, Azithromycin; AMK, Amikacin; VAN, Vancomycin; LVX, Levofloxacin; PB, Polymyxin B; PEX, Pexiganan; ^c^: Interpretive criteria based on CLSI recommendations (2009); ^d^: MSSA, Methicillin-sensitive *Staphylococcus aureus*; ^e^: MRSA, Methicillin-resistant *Staphylococcus aureus*. N/A: Not applicable. *: *P*<0.05, OH-CATH30 vs. the treated drugs or compounds.

### Hemolysis assay

**Figure 2:**
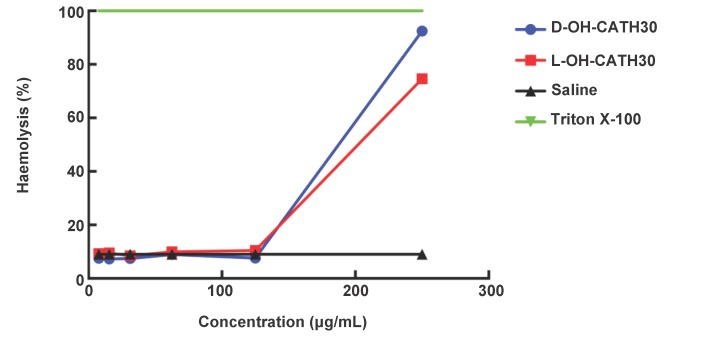
Hemolytic activity of the L- and D- OH-CATH30 peptides

Generally, the peptide and its D- analog showed little difference in hemolytic activity to human red blood cells (Figure 1A). When the dose of L-OH-CATH30 or D-OH-CATH30 was lower than 125 μg/mL, similar hemolysis rates of about 10% were observed, but as the dose increased to 250 μg/mL, high hemolytic activities exceeding 70% for the peptide and 80% for its D- analog were detected (Figure 2).

Peptides at various concentrations were incubated with human red blood cells for 4 h at 37 °C.

### Nitric oxide release

**Figure 3:**
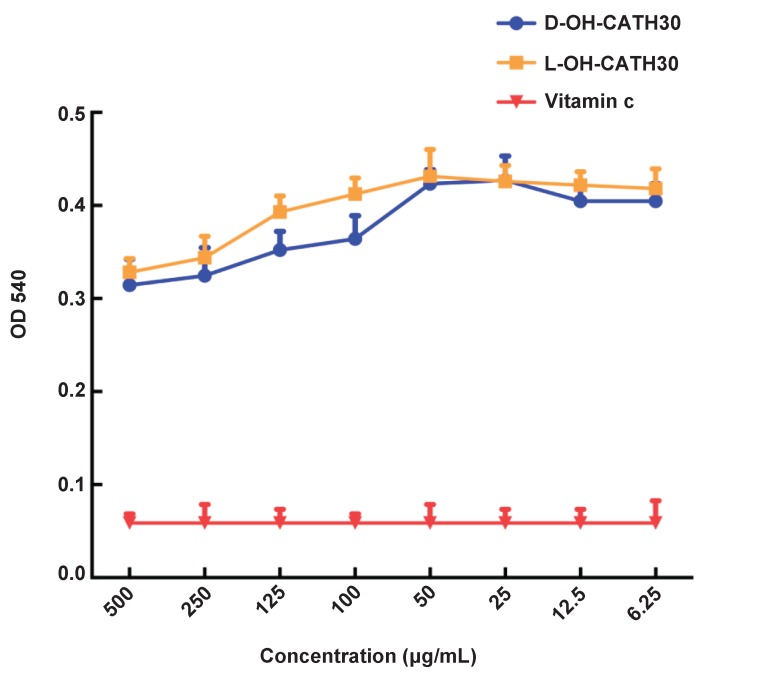
Nitric oxide release induced by L- and D- OH-CATH30 peptides *in vitro*

The peptide and its D- analog displayed steady nitric oxide release when the concentration ranged from 500 μg/mL to 6.25 μg/mL. The amount of nitric oxide released tripled than that in the vitamin C as control group, suggesting that a low concentration of OH-CATH30 stimulated relatively high nitric oxide release (Figure 3).

### IL-6 immunogenicity response

**Figure 4:**
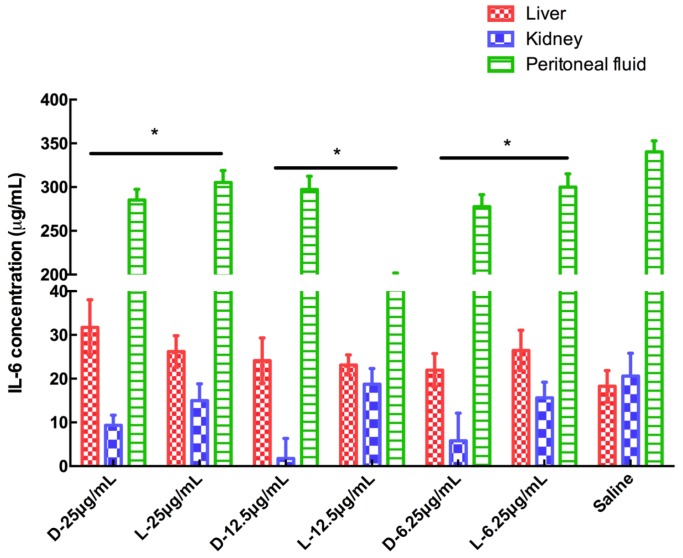
IL-6 induced by L- and D- OH-CATH30 peptides *in vitro*

**Figure 5:**
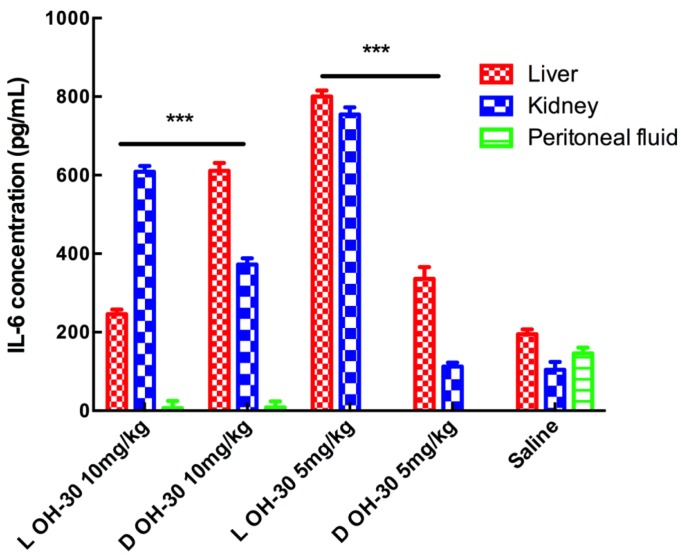
IL-6 induced by L- and D- OH-CATH30 peptides *in vivo*

L-OH-CATH30 is more effective in increasing the IL-6 concentration during an immunogenicity response both *in vitro* and *in vivo* compared with the peptide and its D-analog. Interestingly, OH-CATH30 showed the lowest IL-6 immunogenicity response in the kidney *in vitro*, whereas L-OH-CATH30 showed clear induction of IL-6 in the kidney *in vivo*. By comparison, the peptide and its D- analog promoted IL-6 potently in peritoneal fluid *in vitro* but had virtually no effect on the IL-6 immunogenicity response *in vivo* (Figure 4, Figure 5).

ELISA was used for IL-6 determination. *: *P*<0.05.

Mouse was intraperitoneal injected 2 mL saline 5 h after they were intraperitoneal injected 10 mg/kg and 5 mg/kg the above peptides. ***: *P*<0.001.

## DISCUSSION

Drug resistance in bacteria, in particular the rise of multi-drug resistance, poses a serious threat to human health that urgently requires new drugs with broad efficiency against clinically-encountered bacteria (Leid et al., 2012). AMPs, as novel therapeutic drugs, are being used increasingly to treat infections. In our previous study, OH-CATH30 was found to possess relative normal bactericidal activity and low toxicity *in vivo* and *in vitro* (Li et al., 2012), indicating that it is a competitive candidate for the development of novel antimicrobial agent. 

Here, we tested OH-CATH30 and its D- analog against 584 clinical isolates, including several of drug-resistant pathogens. The data showed that this peptide and its D- analog are broadly active against important medical pathogens including MRSA, MRAB, *S. pneumoniae* and *Pseudomonas* spp. Importantly, this peptide showed high efficacy against Gram- positive bacteria equal to amikacin, and higher efficacy against almost all Gram-negative bacteria tested than that of the peptide-antibiotic polymixin B (shown in Table 1). This peptide also showed good antimicrobial activity against *Acinetobacter* spp., which is primarily associated with nosocomial infections in severely- ill patients in which the management of infections is difficult due to increasing resistance to multiple classes of antibacterial agents (Karageorgopoulos et al., 2008). Our results indicated that OH-CATH30 and its D- analog have higher efficacy against these strains compared with routine antibiotics. 

Usually, searching for new antibiotics, we particularly focus on the cytotoxicity and immunogenicity. Meanwhile, L-OH-CATH30 and D-OH-CATH30 showed low cytotoxicity to eukaryotic cells (red blood cell in our experiments), compared to bacterial cells (their high bactericide activity). They had little difference in hemolytic activity and free radical scavenging ability. IL-6 assay results show that both L-OH-CATH30 and D-OH-CATH30 promoted the release of IL-6 no matter *in vitro* or *in vivo*, which varied in different tissue and treatment doses. It indicated that this peptide could stimulate the immune system to protect against infections *in vitro* or *in vivo*. Furthermore, L-OH-CATH30 and D-OH-CATH30 also showed differences in bactericidal activity against a range of clinical isolates, suggesting that OH-CATH30 containing L- residues is more effective against some pathogens than OH-CATH30 containing D- residues.

## CONCLUSION

In summary, our *in vitro* studies demonstrated that OH-CATH30 and its D- analog are effective against a broad range of clinical isolates. This peptide exerts antimicrobial activity against a multitude of virulent bacteria from various human sources, and has the potential of treating a wide range of bacterial infections, OH-CATH30 is therefore a promising candidate for the development of a new broad-spectrum antimicrobial drug.
